# Microbiome-immune interactions in tuberculosis

**DOI:** 10.1371/journal.ppat.1009377

**Published:** 2021-04-15

**Authors:** Giorgia Mori, Mark Morrison, Antje Blumenthal

**Affiliations:** The University of Queensland Diamantina Institute, Faculty of Medicine, The University of Queensland, Brisbane, Australia; Carnegie Mellon University, UNITED STATES

## Abstract

Tuberculosis (TB) remains an infectious disease of global significance and a leading cause of death in low- and middle-income countries. Significant effort has been directed towards understanding *Mycobacterium tuberculosis* genomics, virulence, and pathophysiology within the framework of Koch postulates. More recently, the advent of “-omics” approaches has broadened our appreciation of how “commensal” microbes have coevolved with their host and have a central role in shaping health and susceptibility to disease. It is now clear that there is a diverse repertoire of interactions between the microbiota and host immune responses that can either sustain or disrupt homeostasis. In the context of the global efforts to combatting TB, such findings and knowledge have raised important questions: Does microbiome composition indicate or determine susceptibility or resistance to *M*. *tuberculosis* infection? Is the development of active disease or latent infection upon *M*. *tuberculosis* exposure influenced by the microbiome? Does microbiome composition influence TB therapy outcome and risk of reinfection with *M*. *tuberculosis*? Can the microbiome be actively managed to reduce risk of *M*. *tuberculosis* infection or recurrence of TB? Here, we explore these questions with a particular focus on microbiome-immune interactions that may affect TB susceptibility, manifestation and progression, the long-term implications of anti-TB therapy, as well as the potential of the host microbiome as target for clinical manipulation.

## Tuberculosis is a globally dominant infection with a long-term burden of antibiotic use

Tuberculosis (TB) persists as one of the top 10 causes of death in the world, with currently an estimated 1.4 million deaths annually [1]. Morbidity and mortality are associated with active TB disease, which is believed to develop in 5% to 10% of individuals that are exposed to and infected by *Mycobacterium* (*M*.) *tuberculosis*. In the majority of individuals, *M*. *tuberculosis* infection is thought to result in clinically asymptomatic latent tuberculosis infection (LTBI). There is currently no standardized test to confirm the presence of viable *M*. *tuberculosis* in individuals with LTBI, and diagnosis is largely based on immunological tests that indicate antigen experience (e.g., skin reactivity to *M*. *tuberculosis* purified protein derivatives (PPD); IFNγ release assays (IGRA) detecting reactivity of CD4^+^ T cells to *M*. *tuberculosis*-specific antigens in whole blood). Of note, there are reports of individuals showing no signs of antigen experience or active TB disease in settings of repeated high exposure and transmission of *M*. *tuberculosis*. While it is difficult to determine how these “resisters” may be protected from productive infection with *M*. *tuberculosis*, a range of innate and adaptive immune mechanisms governed by genetic and epigenetic factors, as well as antigen experience may contribute [2]. It is currently estimated that one quarter of the world’s population is latently infected with *M*. *tuberculosis* [3], with a calculated 5% to 10% lifetime risk of developing active TB [1,4]. Nevertheless, a recent review of human cohort studies undertaken before and after antibiotics became available reemphasized that active TB disease most commonly develops within 1 to 2 years of (confirmed or likely) exposure to *M*. *tuberculosis*. The review of historic data suggested that the risk for active TB beyond 2 years after exposure declines sharply, arguing that reactivation of LTBI might be a much less common event than currently believed and that active TB later in life might result from re-exposure rather than reactivation [5].

First-line anti-TB antibiotics isoniazid, pyrazinamide, and ethambutol are narrow-spectrum, showing little or no activity outside the mycobacterial genus [6], but are often combined with the broad-spectrum antibiotic rifampin, which affects a wide range of Gram-positive and Gram-negative bacteria [1,7]. Indeed, TB antibiotics are being administered to millions of people every year, with up to 780 narrow- and broad-spectrum antibiotic doses administered over a 9-months period [8,9]. This represents one of the longest duration antibiotic regimens used globally. Given the recognized effects that antibiotics have on the composition and function of the host microbiome [10], it is not surprising that conventional TB therapeutic regimens are associated with long-lasting alterations of the gut microbiota in patients and animal models, with impact noted for up to 8 years in a study following patients that were treated for drug-resistant TB (DR-TB) [11–13]. Moreover, significant risk factors for developing active TB, including HIV infection, malnutrition, smoking, alcohol, and diabetes [1,14–17], are associated with both structural and functional changes in the gut microbiota. How these comorbidities, their clinical management and long-term antibiotic use affect the lung microbiome remains poorly understood [12,18–21]. Yet, profound and long-lasting impact on the microbiota is likely to have deleterious consequences for susceptibility and immune control of infectious diseases, including TB.

## The microbiome in health and disease

The colonization of the host by microorganisms begins within minutes of birth or hatching. There is a gradual succession in the diversity and density of these communities, influenced by a myriad of genetic, environmental, and behavioral inputs [22,23]. During those eras of microbiology governed by microscopy and later, culture-based methods, these communities were deemed to be largely comprised of “commensal” microbes: deriving benefits from residing with the host, but with relatively benign and/or unknown impacts on the host itself. The expansion of cultured microbes from different body sites using techniques in anaerobic microbiology helped explain and expand the appreciation of the mutualistic relationships between these communities and their host in terms of structural, metabolic, and immune development [24]. As such, these communities can be considered as the “x-factor” in the genotype x environment x lifestyle interactions governing host response and phenotype. The step advances in nucleic acid sequencing technologies have enabled a phylogenetic and/or gene-based functional assessment of the microbial communities resident at different body sites, and which is commonly referred to as the human microbiome.

By removing the obligatory step of microbial cultivation, a much greater appreciation of the structural and functional dynamics of these communities in the context of health and disease has been developed. In addition to the oral cavity, the microbiota of the large intestine is the most studied compartment of the “human microbiome” [19]. Until recently, microbiome composition was almost exclusively characterized using amplicons produced from the gene encoding 16S rRNA [25]. However, over the last decade, efforts such as the Human Microbiome and integrated Human Microbiome Projects [26] have expanded the scope of investigation to include other regio-specific communities of the human body, the provision of functional as well as taxonomic information via “shotgun metagenomic sequencing” and thereby, a more holistic examination of all 3 domains of life (i.e., Bacteria, Archaea, Eucarya, and their respective viromes) extant (and extinct) in these communities [27–31]. Collectively, these efforts might be summarized into 5 key concepts relevant to our understanding of the roles of the human microbiota in health and disease: First, our microbiota have coevolved with us, drawn from a rather restricted range of the phyla assigned across all 3 domains of life and known to exist in nature. There is a remarkable amount of similarity among the bacterial phyla resident at different body sites, with complexity (and individuality) at different body sites reflected at higher levels of classification [32,33]. Second, this complexity includes a substantial amount of “dark matter” that currently remains biologically uncharacterized at the organismal and genetic level [34]. Third, body sites previously considered to be sterile, such as the healthy lung [35], are now recognized to harbor a variable but nontransient community of microbes considered relevant to sustaining tissue homeostasis with emerging roles in the host defense against pathogenic organisms [36]. Fourth, the advances in food industrialization, medicines, antibiotic use, and hygiene are proposed to impose selective pressures on (at least) the colonic microbiota of Western societies and diminished diversity (“missing microbes”) is linked with the increased incidence of chronic and noncommunicable diseases [37,38]. Indeed, while the definition of a healthy microbiome remains enigmatic, the concept of “dysbiosis” (alterations in measures of microbial diversity and community composition compared to asymptomatic and/or healthy individuals) is now widely considered a hallmark of many chronic and noncommunicable diseases [39,40]. Finally, there are dynamic and bidirectional interactions between the immune system and microbiota with both local and systemic impacts. One example is the multifaceted interplay between the gastrointestinal microbiota and the respiratory tract, coined the gut-lung axis [19]. In this review, we draw on central aspects of these concepts in highlighting the emerging links and implications for TB.

## Microbiota in the *M*. *tuberculosis*-infected host

**Fig 1:**
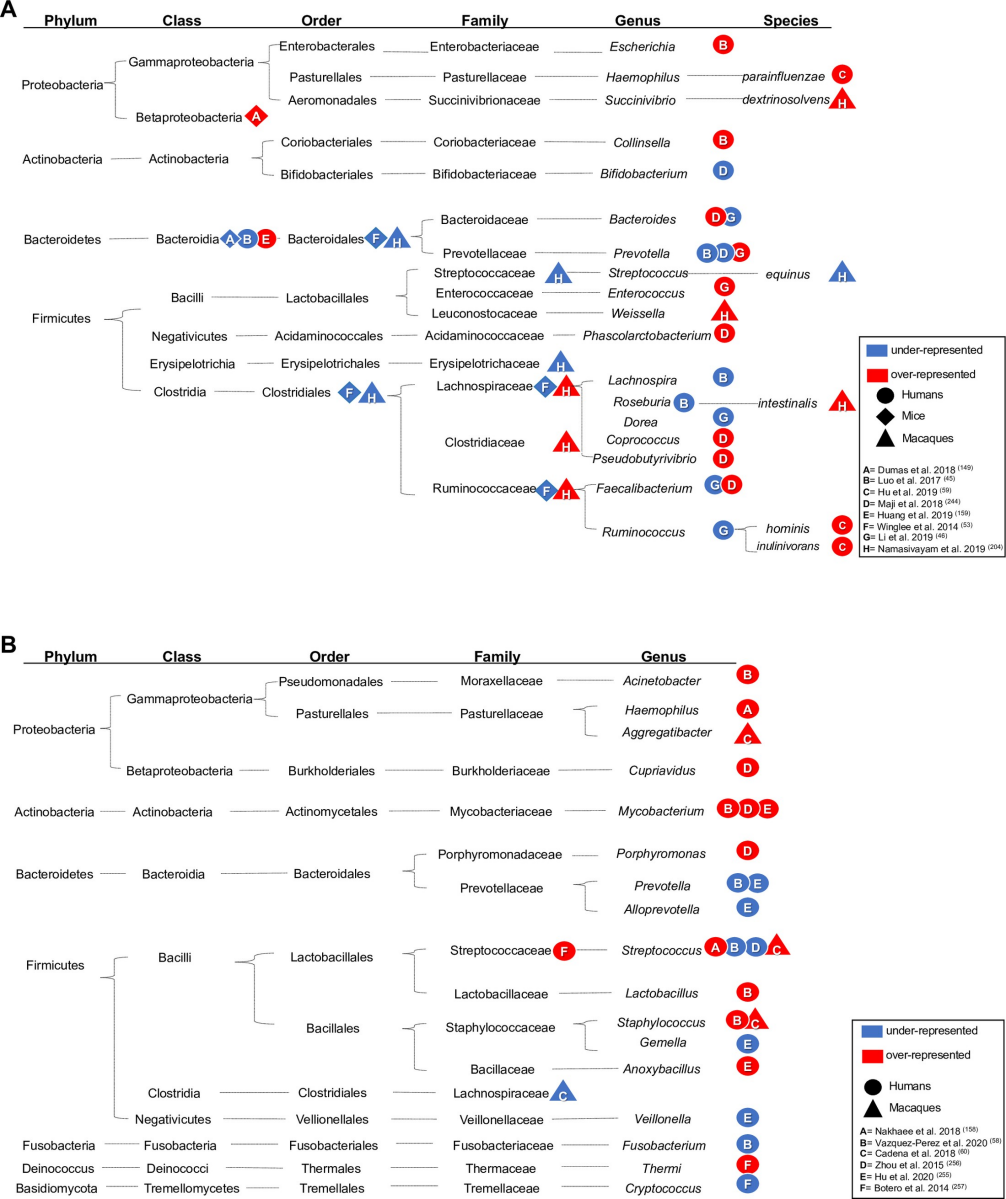
**Alterations in microbiome composition (A = gut; B = respiratory tract) in individuals with active TB compared to controls.** Significantly over- and underrepresented bacteria in the gut (A) and lungs (B) of TB patients (circle), mice (rhombus), or macaques (triangle) models of TB. Taxonomic details are shown, and over- or underrepresentation of the taxonomic level reported by each study is indicated by a red or blue shape, respectively.

Characterization of the microbiome composition of TB patients and the *M*. *tuberculosis*-infected host in animal models has been the subject of significant efforts (Table 1) and has been reviewed in significant detail elsewhere [8,41–44]. Table 1 and Fig 1A and 1B summarize the findings from colonic (fecal) and lung microbiota of humans and animal models of *M*. *tuberculosis* infection compared to noninfected “controls”. In general terms, the fecal microbiota profiles of treatment-naïve, new-onset, and recurrent TB patients consistently show a decrease in bacterial diversity compared to control individuals [45,46]. Phylogenetic integration of the data available through these studies reveals changes to the relative abundances of the bacterial lineages affiliated with the families of Ruminococcaceae and/or Lachnospiraceae (Fig 1A). It is important to note that increased and decreased relative abundance, as well as no significant changes have been reported (Table 1 and Fig 1A), highlighting the challenges posed by integrating data obtained across different host organisms, control populations, and study designs. Nevertheless, these 2 bacterial families of the phylum Firmicutes represent the 2 numerically most abundant groups of Gram-positive bacteria in the human colon [47]. Members of both groups are recognized for their capacity to utilize carbohydrates in simple and polymeric forms and govern the schemes of anaerobic fermentation that produce the short-chain fatty acids (SCFAs) acetate and/or butyrate [48]. Butyrate exerts immunomodulatory effects (discussed below), but it is important to emphasize that members of these bacterial lineages also produce other factors that have been ascribed “anti-inflammatory” capacity [49–51], albeit their impact on host responses to *M*. *tuberculosis* infection, if any, needs to be explored. Moreover, variable changes in the relative abundances of non spore-forming Gram-negative bacterial lineages assigned to the phylum Bacteroidetes (e.g., *Prevotella* and *Bacteroides)* are reported, and relative abundances of Proteobacteria, which, when remarked upon, are increased in *M*. *tuberculosis*-infected individuals (Fig 1A). During anaerobic growth, these latter bacterial groups favor the formation of “mixed acids” including succinate, lactate, formate, but also SCFAs such as propionate and acetate [52]. In addition, structural components in particular the Gram-negative bacterial cell wall component lipopolysaccharide (LPS) can trigger substantial pro-inflammatory responses at local and distant sites if epithelial barrier functions are perturbed (discussed below). Taken together, these findings indicate that *M*. *tuberculosis* infection is associated with a gut “dysbiosis.” While the cause-and-effect relationship between TB and gut dysbiosis is currently unknown, longitudinal analysis of the fecal microbiota in a mouse model suggest that *M*. *tuberculosis* infection causes a significant decrease of the relative abundances of the Lachnospiraceae and Ruminococcaceae families within days of infection [53]. Given that mycobacterial DNA was not detected in fecal samples of infected mice, the selective decrease in bacterial diversity and the dysbiosis observed was unlikely due to the presence of *M*. *tuberculosis* within the gut. These findings suggest that the dysbiosis of the colonic microbiota associated with TB may reflect early alterations in the mucosal immune milieu presented in the gut as a consequence of *M*. *tuberculosis* infection in the lung, and their translation to selective pressures on the colonic microbiota [53]. Importantly, however, whether (transient) changes in the relative abundance of bacterial taxa affects host responses to *M*. *tuberculosis* infection is unknown. In addition, anaerobic growth in the gut is likely to favor metabolic pathways that result in similar classes of metabolites (e.g., SCFAs) across different bacterial taxa. Thus, future studies should aim to combine longitudinal microbiome analyses with transcriptome and metabolome profiling to establish whether changes in the relative abundance of any taxa translate into biologically meaningful changes in the concentrations of immunomodulatory metabolites, and other molecules, at local and distant tissue sites.

**Table 1:** Summary of microbiome studies performed on animal models of TB and TB patients, investigating the impact of *M*. *tuberculosis* infection on the host microbiome.

Impact of *M*. *tuberculosis* infection on the host microbiome						
Location	Specimen	Host and study design	Change in microbiota composition	Effects on the immune system	Sequencing technology and data analysis	Ref
**Gut**	Feces	Newly diagnosed TB patients (NTB, *n* = 19) and recurrent TB patients (RTB, *n* = 18); Healthy controls (*n* = 20)*	Decrease of *Prevotella*, *Lachnospira*, *Roseburia*, and Bacteroidetes in NTB and RTB groups; Enrichment of *Escherichia* and *Collinsella* genera in RTB.	*Lachnospira* and *Prevotella* directly correlated with CD4^+^ cell counts in peripheral blood of NTB and inversely correlated with RTB.	16S rRNA gene amplicon (Illumina) sequencing; Greengenes database^Δ^; Quantitative Insights into Microbial Ecology (QIIME Version 1.7.0°)	[45]
	Feces	TB patients who did not receive antibiotics 1 month prior to enrollment (*n* = 18); healthy controls (*n* = 18)	Decrease of *Faecalibacterium*, *Bacteroides*, *Ruminococcus*, and *Dorea*; increase of *Enterococcus* and *Prevotella* genera.	n.d.	16S rRNA gene amplicon (Illumina) sequencing; Greengenes database^Δ^; (QIIME v 1.9.1°)	[46]
	Feces	TB patients (*n* = 6) (fecal samples collected before the start of treatment); healthy individuals (*n* = 6)	Increase of *Faecalibacterium*, *Coprococcus*, *Phascolarctobacterium*, *Bacteroides*, and *Pseudobutyrivibrio*; decrease of *Prevotella*, *Bifidobacterium*	n.d.	16S rRNA gene amplicon (Illumina) sequencing; Greengenes database^Δ^; (QIIME v 1.8°)	[244]
	Feces	TB patients (*n* = 46); healthy individuals (*n* = 31)	Presence of *Haemophilus parainfluenzae*, *Roseburia inulinivorans*, and *Roseburia hominis* in TB patients but not controls	n.d.	Shotgun metagenomic Illumina sequencing; Metaphlan2 (species abundance)	[254]
	Feces	TB patients (*n* = 25); LTBI patients (*n* = 32); healthy individuals (*n* = 23)	A higher relative abundance of Bacteroidetes concurrent with low Firmicutes/Bacteroidetes ratio in active TB and LTBI	Positive association of Bacteroidetes and polymorphonuclear neutrophils in TB and LTBI patients; concurrent increase of pro-inflammatory cytokines (IL-6 and IL-1B) and low relative abundance of Bifidobacteriaceae in TB patients	16S rRNA gene amplicon (Illumina) sequencing; Greengenes database^Δ^; QIIME°	[159]
	Feces	Female Balb/c mice (*n* = 5) infected with *Mtb* CDC1551 or *Mtb* H37Rv; preinfection samples from each group as control (*n* = 3)	Decrease of Clostridiales (Lachnospiraceae, Ruminococcaceae families) and Bacteroidales orders.	n.d.	16S rRNA gene amplicon (454) pyrosequencing sequencing; Silva database^Δ^; QIIME°	[53]
	Feces	Female C57BL/6 mice treated with a cocktail of broad-spectrum antibiotics ceased 2 days before *Mtb* infection; control group mice w/o Abx treatment; stool samples collected after intranasal *Mtb H37Rv* infection (*n* = 4–14 mice/group)	Decrease of Bacteroidetes and Firmicutes; increase of Betaproteobacteria	Decrease in MAIT cells and IL17A in the lungs and increased susceptibility to *Mtb*	RT-qPCR was performed using phylum-specific primers	[149]
	Feces	Female C57BL/6J-CD45a(Ly5a) mice (*n* = 3–5), 4–8 weeks old, infected with *Mtb* H37Rv; uninfected age-matched control (*n* = 3–5), repeated sampling over 20 weeks of infection	Decreased relative abundance of Clostridiales; increased Bacteroidales; although neither significant by 20 weeks	n.d.	16S rRNA gene amplicon (Illumina) sequencing; custom reference database built from the NCBI 16S rRNA gene sequence and taxonomy database (version May 2016^Δ^; QIIME v 1.9.1°)	[11]
	Feces	Rhesus macaques (*n* = 4–6) infected with *Mtb* Erdman	Families Lachnospiraceae, Ruminococcaceae, and Clostridiaceae significantly increased in animals with severe disease; members of the family Streptococcaceae, Erysipelotrichaceae, and the Bacteroidales RF16 and Clostridiales vadin B660 groups were decreased in the same group. *Roseburia intestinalis*, *Succinivibrio dextrinosolvens*, certain Ruminococcaceae, and *Weissella* were enriched, and *Streptococcus equinus* was decreased in some or all animals with severe disease.	n.d.	16S rRNA gene amplicon (Illumina) sequencing; Silva database^Δ^; QIIME2/ DADA2°; Shotgun metagenomics with NextSeq 500 platform	[204]
**Respiratory tract**	BAL	Pulmonary TB patients (TB) (*n* = 6); healthy controls (*n* = 10)	Decrease of *Streptococcus*, *Prevotella*, *Fusobacterium*; increase of *Lactobacillus*, *Acinetobacter*, *Mycobacterium*, and *Staphylococcus* genera.	n.d.	16S rRNA gene amplicon (Illumina) sequencing; (QIIME v 1.8°)	[58]
	BAL	*Mtb*-positive (MTB+, *n* = 70) and *Mtb*-negative (MTB−, *n* = 70) TB patients^#^	*Mycobacterium* and *Anoxybacillus* genera highly abundant in MTB+; MTB− microbiota enriched with *Prevotella*, *Alloprevotella*, *Veillonella*, and *Gemella* genera.	n.d.	16S rRNA gene amplicon (Illumina) sequencing; Silva database^Δ^; Mothur (v 1.35.1°)	[255]
	BAL	TB patients (*n* = 10); healthy controls (*n* = 5)	Presence of the 4 important genus of lung microbiota (*Streptococcus*, *Neisseria*, *Veillonella*, and *Haemophilus*)	Frequency of *Streptococcus* directly correlated with TB; frequency of *Haemophilus* in TB patients is correlated with expression level of *T-bet* gene (Th1 immune response)	Lung microbiota was detected through culture methods.	[158]
	BAL	TB patients (*n* = 32); healthy controls (*n* = 24)	*Cupriavidus* dominance and decrease of *Streptococcus* in TB patients; wide distribution of *Mycobacterium* and *Porphyromonas* in TB patients	n.d.	16S rRNA gene amplicon (454) pyrosequencing; Ribosomal Database Project (RDP)^Δ^; Fast UniFrac°	[256]
	nasal, oropharynx, sputum samples	TB patients (*n* = 6); healthy controls (*n* = 6)	Abundance of Thermi phylum and unclassified sequences belonging to the Streptococcaceae family in TB patients; decrease of the genus *Cryptococcus* in TB patients	n.d.	16S rRNA gene and ITS amplicon (454) pyrosequencing; Greengenes database^Δ^; QIIME (v 1.6°)	[257]
	OWs, BALs, bronchoscope control samples	Cynomolgus macaques (*n* = 26) infected with *Mtb* Erdman	Increase of *Aggregibacter*, *Staphylococcus*, *Streptococcus*, and the unculturable Candidate division SR1 bacteria; decrease of Lachnospiraceae	n.d.	16S rRNA gene amplicon (Illumina) sequencing; Greengenes database^Δ^; QIIME°	[60]

*NTB, no more than 1 week anti-TB treatment; RTB, previously treated and declared as cured prior to recurrence.

^#^No healthy individuals recruited as controls, positive *M*. *tuberculosis* (*Mtb*) detection determined by a combination of sputum smear, culture, RT-PCR, and GeneXpert.

^Δ^Taxonomic assignment.

°Operational Taxonomic Units (OTUs) analysis.

BAL, bronchoalveolar lavage; LTBI, latent tuberculosis infection; n.d., not determined; NTB, newly diagnosed TB patients; OW, oral wash; RTB, recurrent TB patients; RT-qPCR, quantitative reverse transcription PCR; TB, tuberculosis.

As reflected in Table 1, the studies of the lung microbiota in TB patients and model organisms are fewer and often represent findings obtained from a relatively small number of individuals. Sputum samples have been commonly used to assess the lung microbiome in TB patients [54,55]. However, potential contamination of these samples with bacterial genera typically present in the oropharyngeal microbiota (e.g., *Prevotella*, *Bulleidia*, and *Atopobium*) [18] needs to be considered [56,57]. Alternatively, samples collected via bronchoalveolar lavage (BAL) require more invasive collection methods but are beginning to provide insight into the microbiota of the lower respiratory tract of humans [58]. The largest study to date used BAL to characterize the lung microbiota of human patients with respiratory symptoms and abnormal imaging results, with and without confirmed *M*. *tuberculosis* infection [59]. The relatively diverse microbial community (e.g., *Streptococcus* and *Prevotella*) in patients without *M*. *tuberculosis* [59] contrasted the BAL microbiota of TB patients, which was dominated by *M*. *tuberculosis*. This highlights potential challenges for the precise annotation of the TB-associated lung microbiota when using 16S rRNA gene profiling [59]. Nevertheless, longitudinal 16S rRNA-based analyses of oral washes, BAL, and bronchoscopy samples in macaques experimentally infected with *M*. *tuberculosis*, revealed increased microbial diversity early after infection (1 month), with the relative abundances of *Aggregatibacter*, *Streptococcus*, and *Staphylococcus* genera elevated by 4 months post infection, and the relative abundances of members of the Lachnospiraceae family being significantly decreased [60]. The magnitude of alterations between individual animals were highly heterogenous, which was discussed to possibly reflect genetic makeup of the individual hosts, previous exposure to infection and treatment, and the heterogenous nature of *M*. *tuberculosis* infection in macaques [60]. Indeed, the caveats highlighted by the authors of this study are reflective of shortcomings of most microbiome research to date, which historically has been undertaken as a part of observational and cross-sectional studies. This has led to calls for the utilization of more rigorous study design in both animal models and clinical studies, and the pursuit of multinational and/or multicultural frameworks to enhance demonstration of causality and progress towards clinical outcomes [61–64]. For instance, longitudinal analyses in a defined experimental setting will be vital for better characterizing microbiome dynamics during *M*. *tuberculosis* infection, and whether these result from microbial interactions within the niche, or as a consequence of mucosal (and peripheral) immune responses to *M*. *tuberculosis* infection. As the importance of microbiome composition of the respiratory tract for susceptibility to infections is emerging [65], constrains imposed by sample type and sequencing approaches will need to be overcome by standardized methods that subtractively enrich microbial DNA from BAL samples, to advance the application of shotgun metagenomic sequencing to provide a more holistic and nonbiased assessment of microbial communities in respiratory health and disease [66,67].

## Impact of TB antibiotics treatment on the host microbiome

**Fig 2:**
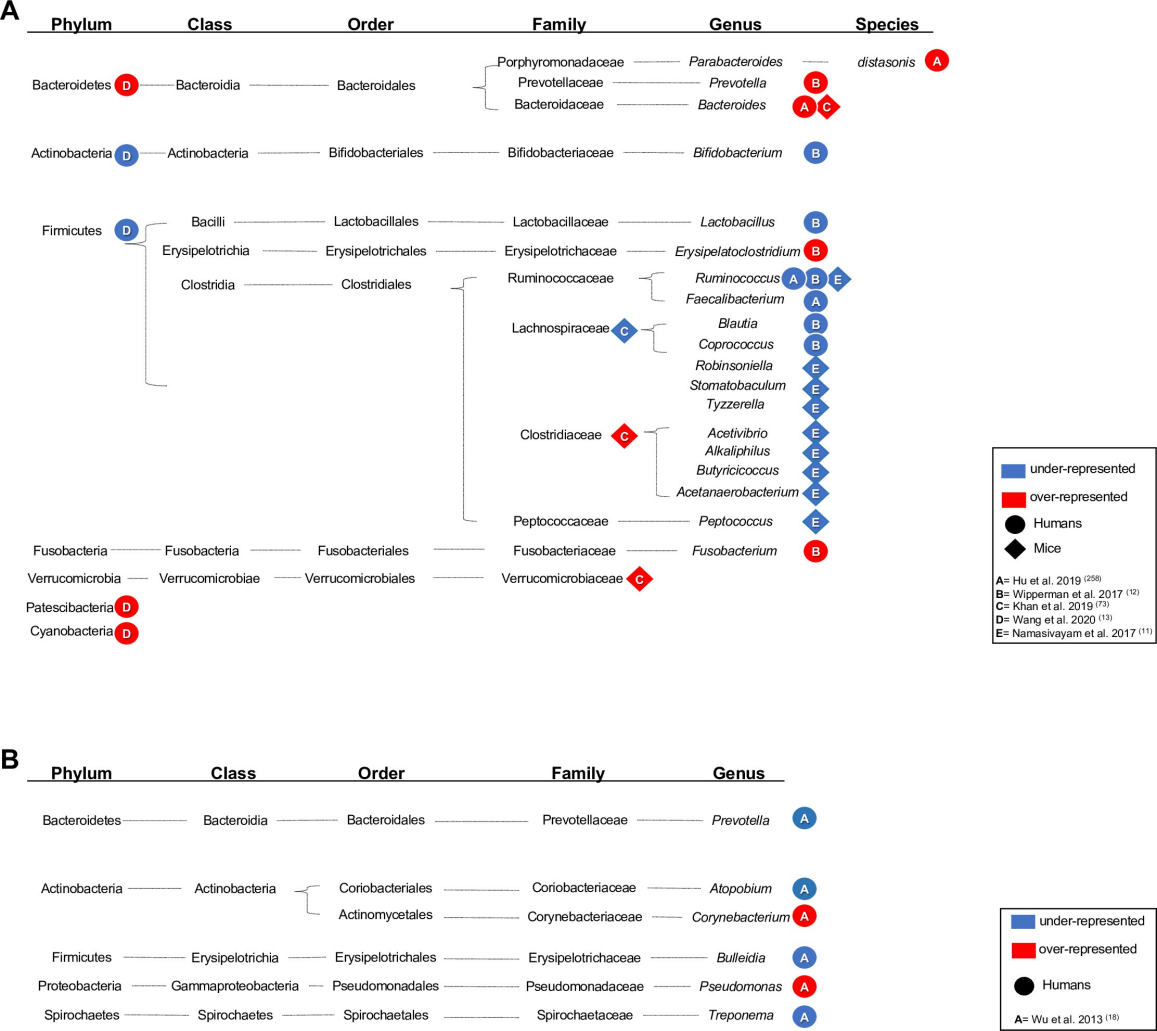
**Alterations in microbiome composition (A = gut; B = respiratory tract) of patients upon TB antibiotics treatment.** Significantly over- and underrepresented bacteria in the gut (A) and lungs (B) of TB patients (circle), mice (rhombus), or macaques (triangle) models of TB undergoing therapy for drug-sensitive or multidrug-resistant TB. Taxonomic details are shown, and over- or underrepresentation of the taxonomic level reported by each study is indicated by a red or blue shape, respectively.

The phenotypic and genetics basis of drug resistance in *M*. *tuberculosis* is one of the most significant constraints to improving the clinical management of TB [68]. Treatment regimens for drug-sensitive TB (6 to 9 months) and drug-resistant TB (up to 2 years) are protracted [1]. Antibiotic use disrupts both the composition and total abundance of the microbiota. Whereas there is a limited number of studies addressing this in TB patients and mouse models of *M*. *tuberculosis* infection, the results to date indicate that TB antibiotics have a long-lasting impact on the gut microbiome composition [11–13,42–44]. Table 2 summarizes cross-sectional studies in humans and mouse models that have reported effects of TB antibiotics on the microbiota, with Fig 2 providing a phylogenetic integration of the findings to date. A common theme is an antibiotic-induced dysbiosis, with depleted populations of Gram-positive bacteria assigned to the Ruminococcaceae and Lachnospiraceae.

**Table 2:** Summary of microbiome studies performed on animal models of TB and TB patients, investigating the impact of anti-TB treatment on the host microbiome.

Effects of anti-TB treatment on the host microbiome composition							
Location	Specimen	Host	Treatment	Change in microbiota composition	Effects on the immune system	Sequencing technology and data analyisis	Ref
**Gut**	Feces	LTBI (*n* = 10), TB (*n* = 28) TB patients with 1-week anti-TB therapy (TB1, *n* = 13), TB patients with 2-week anti-TB therapy (T2, *n* = 10, cured TB patients (TBc, *n* = 10); healthy individuals (*n* = 13)	INH, RIF, EMB, and PZA	Decrease of *Ruminococcus* and *Faecalibacterium*. Increased abundance of *Bacteroides* species and *Parabacteroides distasonis* in all the treatment groups.	n.d.	16S rRNA gene amplicon (Illumina) sequencing; Ribosomal Database Project (RDP) ^Δ^; Mothur v.1.36.1°	[258]
	Feces	LTBI (*n* = 25), TB treatment (*n* = 19), cured TB patients (*n* = 19); individuals without *Mtb* infection (IGRA-) as controls (*n* = 50)	INH, RIF, EMB, and PZA	Enrichment of *Erysipelatoclostridium*, *Fusobacterium*, and *Prevotella*; decrease of *Blautia*, *Lactobacillus*, *Coprococcus*, *Ruminococcus*, and *Bifidobacterium* in the TB treatment group. Depletion of *Bacteroides* and overabundance of *Faecalibacterium*, *Eubacterium*, and *Ruminococcus* in cured TB group: *Enterobacter cloacae*, *Phascolarctobacterium succinatutens*, *Methanobrevibacter smithii*, *Bilophila*, and *Parabacteroides* are biomarkers of cured TB patients.	n.d.	16S rRNA gene amplicon (Illumina) sequencing; NCBI refseq_rna database with custom scripts^Δ^; QIIME°/ Shotgun metagenomic Illumina sequencing; Metaphlan2 (microbial species abundances) and HUMAnN2 (functional pathways)	[12]
	Feces	MDR-TB treatment group (*n* = 6) and untreated controls (*n* = 26); MDR-TB recovered group (*n* = 18) and untreated control (*n* = 28)	MDR-TB treatment	Bacteroidetes, Cyanobacteria, and Patescibacteria are biomarkers for the recovered group: decrease of Actinobacteria and Firmicutes; increase of Bacteroidetes in recovered group.	n.d.	16S rRNA gene amplicon (Illumina) sequencing; RDP classifier (v 2.2)^Δ^; Mothur°	[13]
	Feces	6–10 weeks old C57BL/6 mice (*n* = 5) infected with *Mtb* H37Rv; fecal samples collected prior to the treatment as baseline (*n* = 5)	RIF or INH + PYZ	Expansion of *Bacteroides*, Verrucomicrobiaceae, and decrease in Lachnospiraceae in RIF-treated samples; increase of Clostridiaceae in INH/PYZ-treated mice.	Expression levels of MHCII and production of TNFα and IL-1β significantly reduced after *M*. *tuberculosis* infection. Alveolar macrophages more permissive for intracellular *M*. *tuberculosis* replication.	16S rRNA gene amplicon (Illumina) sequencing; Microbiome Analyst web application (community diversity profiling and statistical analysis)	[73]
	Feces	4–8-week-old C57BL/6J-CD45a(Ly5a) female mice (*n* = 3–5) infected with *M*. *tuberculosis* H37Rv; uninfected age-matched control (*n* = 3–5)	INH, RIF, and PZA + INH and RIF	Decrease of genera *Acetivibrio*, *Robinsoniella*, *Alkaliphilus*, *Stomatobaculum*, *Butyricicoccus*, *Acetanaerobacterium*, *Tyzzerella*, *Ruminococcus*, and *Peptococcus* (all belonging to class Clostridia, phylum Firmicutes).	n.d.	16S rRNA gene amplicon (Illumina) sequencing; custom reference database built from the NCBI 16S rRNA gene sequence and taxonomy database (version May 2016)^Δ^; QIIME (v 1.9.1°)	[11]
**Respiratory tract**	Sputum samples and throat swab samples	New TB group (N-TB, *n* = 25): patients, cured new TB patients (C-TB, *n* = 20), recurrent TB group (*n* = 30), treatment failure group (*n* = 20); healthy individuals (*n* = 20)	mix of DS-TB and MDR-TB treatments	*Pseudomonas* abundance in TB treatment failure patients or recurrent TB than in new or cured TB patients; *Prevotella*, *Bulleidia*, *Atopobium*, and *Treponema* decrease in recurrent TB patients than new TB group; increased *Corynebacterium* abundance in recurrent TB than treatment failure TB.	n.d.	16S rRNA gene amplicon (454) pyrosequencing; Greengenes database^Δ^; QIIME (v 1.5.0°)	[18]

^Δ^Taxonomic assignment.

°Operational Taxonomic Units (OTUs) analysis.

DS-TB, drug-susceptible TB; LTBI, latent tuberculosis infection; MDR-TB, multidrug-resistant TB; n.d., not determined; TB, tuberculosis.

It is increasingly appreciated that commensal bacteria can confer a form of colonization resistance against nonresident species including pathogens, via competition for metabolic and/or spatial niches, as well as their production of bioactive molecules that can directly inhibit/suppress the growth of susceptible microbes [69]. The sustained use of antibiotics for recalcitrant *Clostridioides difficile* infection often results in long-term failure of antibiotics to control this infection [69], and this has been used to exemplify how chronic antibiotic use might be a risk factor for reinfection with *M*. *tuberculosis* [70,71]. Indeed, long-term impact of TB antibiotics was indicated by a recent study reporting preferential loss of T cell reactivity to *M*. *tuberculosis*-derived epitopes that showed similarities with microbiota species [72]. In a mouse model, TB antibiotics altered gut microbiota composition and affected the immune responses to *M*. *tuberculosis* infection [73], alluding to the multidimensional complexity of the interplay between resident microbiota at the time of *M*. *tuberculosis* infection and the quality of the immune response. Understanding of how prolonged antibiotic use affects predisposition to recurrent TB and/or reinfection is an important area of future investment. Notwithstanding the limits of current studies (e.g., cohort size, mode of sampling), anti-TB antibiotic regimens exert selective pressure and reorganization of the gut and/or lung microbiota with profound and long-lasting effects. Knowledge of the functional implications of these alterations via the gut-lung axis on host immune response are emerging. The following sections examine the physiological and metabolic cues arising from the gut (and lung) microbiota with implications for host susceptibility or resistance to the clinical manifestations of *M*. *tuberculosis* infection.

## Microbiome-immune crosstalk and host control of *M*. *tuberculosis*

**Fig 3:**
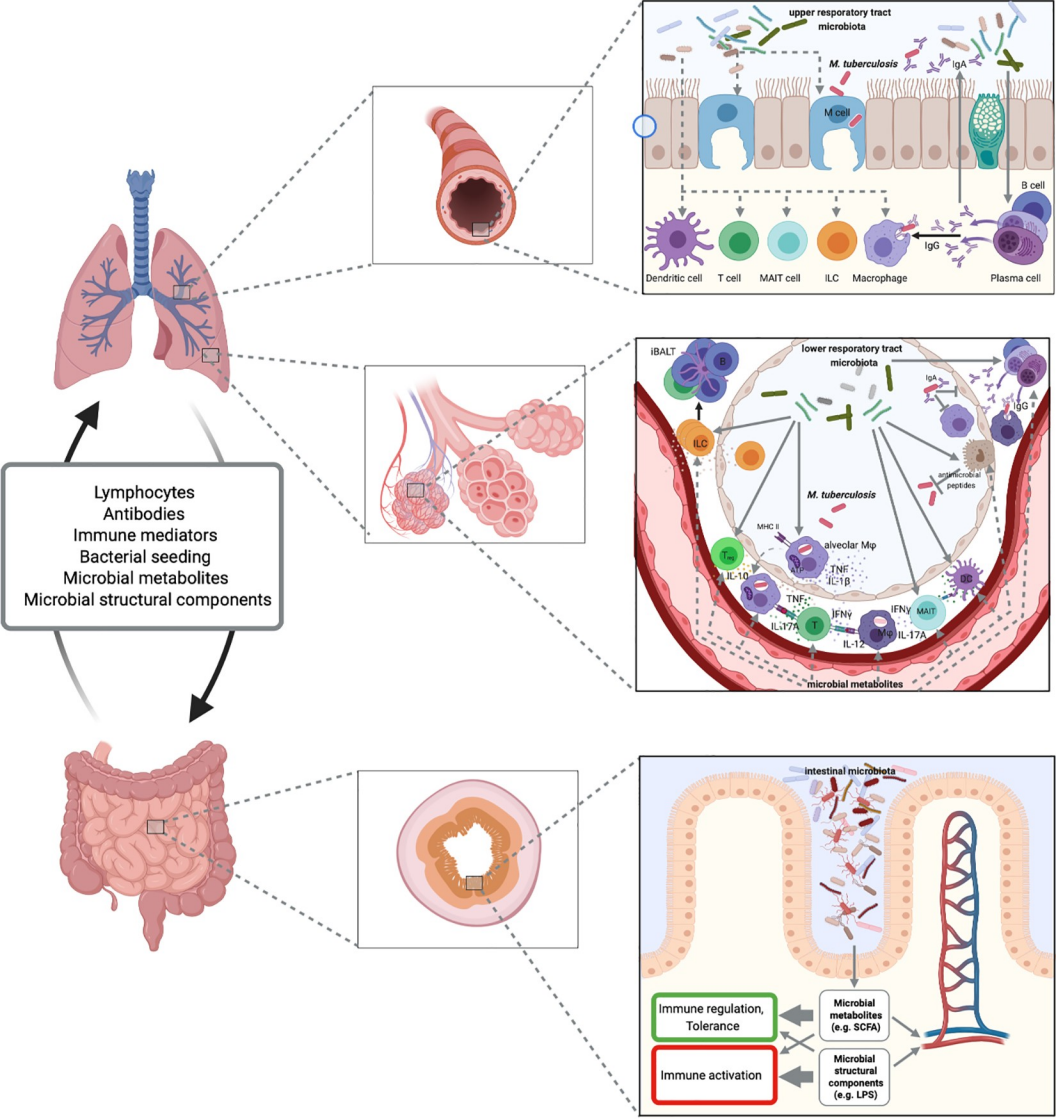
Proposed microbiome-immune interactions in *M*. *tuberculosis* infection. Microbiota of the upper and lower respiratory tract may define epithelial barrier integrity, M cell frequency, antimicrobial defense, composition, and functionality of innate and adaptive immune mechanisms. Through the gut-lung axis, the microbiota of the intestinal tract influences barrier and immune functions in the periphery and at sites of *M*. *tuberculosis* infection. Fig 3 was created with BioRender.

Bioactive metabolites are a key element of the crosstalk between the host and its microbial collective. Such metabolites arise from microbial metabolism (e.g., vitamins) as well as microbe-facilitated modulation of host- or dietary-derived metabolites (e.g., bile acids, SCFAs) [74]. Significant focus to date has been on the metabolic capacity of the gut microbiome, with evidence for impact on immune functions at distant sites, including the lung via the gut-lung-axis [75] (Fig 3). Here, we focus on the emerging concepts of direct and indirect contributions of the host microbiome to host defense mechanisms against *M*. *tuberculosis* infection [44].

### Epithelial barriers and innate immunity

#### Epithelial cells

The main route of *M*. *tuberculosis* entry into the human host is transmission via aerosol droplets. The size of *M*. *tuberculosis*-containing droplets allows entry into the alveoli of the lower respiratory tract where the bacteria encounter respiratory epithelium, alveolar macrophages, and resident microbiota. The roles of alveolar epithelial cells in the host defense against *M*. *tuberculosis* are incompletely understood. *M*. *tuberculosis* has been found in cells of the alveolar epithelium in humans, and infected alveolar epithelial cells in vitro in some but not all studies [76–80]. Transmigration of infected alveolar macrophages from the alveolar space across the epithelium into the interstitium enables engagement of interstitial and recruited inflammatory macrophages, a process important for control of *M*. *tuberculosis* [81]. While the importance of the gut microbiota in maintaining gut epithelial integrity and barrier functions is well established [82–85], it is unknown whether microbiota-epithelial interactions shape alveolar macrophage transmigration or macrophage recruitment to sites of *M*. *tuberculosis* entry. Pulmonary epithelial cellular defense mechanisms are directly responsive to microbiota-derived SCFAs [86]. Whether production of antimicrobial peptides (AMPs) upon encounter with *M*. *tuberculosis* [87,88], is shaped by the lung-resident, or remote, microbiota, will be important to determine as this bears relevance to host defense against *M*. *tuberculosi*s, and bacterial pathogens more generally. Moreover, microfold (M) cells in the upper respiratory tract have been suggested to act as entry points for *M*. *tuberculosis* across the epithelial barrier into mucosa-associated lymphoid tissues, which may result in extrapulmonary manifestation of *M*. *tuberculosis* (e.g., cervical lymphadenopathy in the absence of evidence for pulmonary TB) [89,90]. This process has been reported to be facilitated by interactions between the *M*. *tuberculosis* virulence factor EsxA and scavenger receptor B1 on M cells in the airway epithelium [91]. With microbiota composition implicated in M cell density and functionality in the gut [92], microbiota contributions to airway M cell functions remain to be elucidated, including implications for *M*. *tuberculosis* infection in the antibiotic-naïve or antibiotic-experienced host.

Host-microbiota interactions are critical in governing tissue homeostasis at sites of close contact as well as distant sites. Yet, microbial dysbiosis and compromised but barrier functions, e.g., in the context of chronic inflammation and antibiotics treatment, have been implicated in inflammation and metabolic dysfunction at distant sites. This is driven at least in part through innate immune activation of macrophages and other innate immune cells by microbiota-derived bacterial products such as LPS [83,93–96] (Fig 3). Some studies have questioned whether epithelial functions in the gut are altered in TB patients and how this might affect pharmacodynamics of TB antibiotics and have returned varying results [97–101]. With long-term antibiotics regimens and sustained alterations of the gut microbiota, it is relevant to query if and how the integrity of epithelial barriers (e.g., gastrointestinal and respiratory tract) is affected in TB patients during and after treatment, and whether this has long-term consequences for tissue and organ homeostasis, immune functions, metabolism, cognition, and behavior [83].

#### Macrophages

Macrophages are major host cells for intracellular *M*. *tuberculosis*, and bacterial interference with macrophage antimicrobial defense mechanisms enable intracellular persistence and replication [102]. The immune-regulatory and metabolic phenotype of alveolar macrophages, as well as ready availability of nutrients key to intracellular *M*. *tuberculosis* survival have been implicated in facilitating the exponential intracellular replication of *M*. *tuberculosis* within alveolar macrophages for several days post infection [81]. The airway microbiota has been implicated in defining alveolar macrophage functions [75,103], including during *M*. *tuberculosis* infection [73]. Infection of mice with *M*. *tuberculosis* after a course of treatment with the TB antibiotics isoniazid and pyrazinamide for 8 weeks resulted in slightly higher lung bacterial burden. This was accompanied by an altered phenotype of alveolar macrophages, including diminished MHCII expression, TNF and IL-1β production, as well as cellular respiration and ATP production [73]. Alveolar macrophages derived from such antibiotic-treated mice were diminished in their ability to control intracellular *M*. *tuberculosis* replication in ex vivo cultures. The authors linked functional dysbiosis to these outcomes, which were reversed by fecal microbiota transfer (FMT). It is interesting to note that the antibiotic-driven phenotypic alteration of alveolar macrophages was not inducible in in vitro culture in the presence of isoniazid and pyrazinamide but required the in vivo tissue context [73], suggesting that alveolar macrophage phenotypic imprinting required tissue- and/or microbiome-derived factors. In this context, it is noteworthy that in in vitro cultures of PBMC, the presence of lactic acid bacteria has been reported to enhance *M*. *tuberculosis*-induced IFNγ production and promoted IFNγ-driven macrophage antimicrobial defense mechanisms [104]. Thus, positioning the microbiota as one of the likely sources of cues that define alveolar macrophage functions related to antimicrobial defense, inflammation, and engagement of adaptive immunity is important for our understanding of early host control of *M*. *tuberculosis* infection with implications for developing active disease or LTBI.

#### Innate and innate-like lymphoid cells

Microbial products, including metabolites, distinctly guide development and functions of innate and innate-like lymphocytes. Conversely, the localization of innate and innate-like lymphoid cells to mucosal sites directs the composition and spatial distribution of the microbiota [105]. SCFAs such as acetate, propionate, and butyrate are the most abundant bacterial products derived from commensal bacterial fermentation of dietary fibers in the intestine and have been found to regulate cellular metabolism and exert potent immune-regulatory functions [106,107]. SCFAs instruct the proliferation and function of group 3 innate lymphoid cells (ILC3) [108], which play central roles in immune responses at mucosal and epithelial sites, including the lung [109]. Control of *M*. *tuberculosis* infection is critically dependent on intact IL-12 and IFNγ signaling, and IFNγ-mediated protection is largely attributed to adaptive T cell responses [110]. However, more recently, contributions of innate and innate-like lymphoid cells have been unveiled.

Based on their cytokine expression profiles, ILCs are categorized into group 1, including natural killer (NK) cells and noncytotoxic type 1 ILCs (IFNγ, TNF), group 2 (IL-4/5/13), and group 3 (IL-17/22) [111].

IFNγ-expressing NK cells have been described to accumulate in the pleural fluid of patients with TB pleurisy [112]. Individuals with LTBI exhibited increased numbers of circulating NK cells in peripheral blood and these cells exhibited increased cytotoxic capacity associated with high expression of granzyme B and perforin [113], and accumulation of CD27^+^ NK cells in the lung has also been associated with LTBI in nonhuman primates [114]. In contrast, circulating NK cells were markedly decreased in peripheral blood of patients with active TB [113]. NK cells have been reported to contribute to CD8^+^ T cell responses, and lyse mycobacteria-infected monocytes, macrophages, as well as regulatory T cells expanded in the presence of mycobacterial antigens [115–117]. Patients with active TB exhibit diminished proportions of type 1, 2, and 3 ILCs, but not NK cells, in peripheral blood [118], which is thought to be a result of ILC accumulation in infected lungs, as has been shown for mice infected with *M*. *tuberculosis* or *Mycobacterium bovis* bacille Calmette-Guérin (BCG) [118,119]. Transcriptome analyses of ILC2s and ILC3s isolated from lungs of TB patients revealed distinct immune signatures [118], suggesting specific functional contributions. Early studies in mice indicated that deficiency in T and B lymphocytes as well as ILCs (RAG2^−/−^ γc^−/−^) resulted in higher susceptibility to *M*. *tuberculosis* infection compared to T and B cell deficiency (RAG2^−/−^), which was attributed to IL-12-driven IFNγ production by innate lymphocytes [120]. More recently, specific contributions of group 3 ILCs to host control of *M*. *tuberculosis* early during infection have emerged, specifically in the formation of inducible bronchus-associated lymphoid tissue (iBALT) [118], which is associated with a degree of host protection early during *M*. *tuberculosis* infection [121].

Due to the intimate connection between microbiota and ILCs, many questions arise from these recent observations, including: Are ILC3 contributions to immune control of *M*. *tuberculosis* shaped by the metabolic capacity of the microbiome (e.g., dynamics and relative abundance of SCFA at mucosal sites and in the periphery [108]? Do (myco)bacteria-derived components or TB antibiotics direct ILC3 functions, e.g., through engagement of arylhydrocarbon receptor (AhR) [122,123], a ligand-dependent transcription factor that governs ILC3 functions [124]? Are microbiota-derived metabolites that drive IL-22 production at mucosal sites (e.g., tryptophan derivatives) [125] linked to the host control of *M*. *tuberculosis* attributed to type 3 ILC and IL-22 [118,126,127]? Does plasticity within type 1 ILC (i.e., conversion of NK cells to type I ILCs) occur during *M*. *tuberculosis* infection, similar to what has been described recently in the context of *Toxoplasma gondii* infection [128] and tumor immune evasion [129]? Is ILC functionality at the sites of *M*. *tuberculosis* infection reflective of the ILC composition detectable in peripheral blood and do alterations in the periphery indicate relevance to host control, e.g., as discussed for NK cell dynamics in active TB versus LTBI and healthy controls [113,130,131]?

#### MAIT cells

Innate-like lymphocytes, including mucosa-associated invariant T cells (MAIT), natural killer T cells (NKT), and γδ T cells recognize microbially derived nonpeptide antigens via semi-invariant T cell receptors (TCRs) resulting in cytokine production and/or cytotoxic activity. Among these, MAIT cell development has been closely linked to the presence of the microbiota driven by thymic presentation of bacteria-derived antigen [132–135], although microbiota-independent MAIT cell development during embryogenesis has also been reported [136]. MAIT cells are abundant in barrier tissues and at mucosal sites, including the lung, apart from representing up to 10% of circulating human T cells [137]. The evolutionary conserved MAIT cell TCRs have been shown to recognize the vitamin B2 precursor metabolite, 5-(2-oxopropylideneamino)-6-D-ribitylaminouracil (5-OP-RU), presented by the MHC-1-like molecule MR1 [138,139]. In addition, IL-18 and IL-12 can drive antigen-independent activation of MAIT cells [140]. TCR-mediated MAIT cell effector functions include cytokine production (predominantly IL-17A by MAIT cells in mice and human tissues; IFNγ, TNF in human blood), cytotoxicity against cells that present antigen via MR1, and bystander activation of dendritic cells [137].

Peripheral blood MAIT cell numbers are significantly diminished in TB patients [141–146] and have been noted to negatively correlate with TB disease severity [143]. A TB household contact study reported that MAIT cells in peripheral blood show signatures of activation [147]. Whereas MAIT cell accumulation in infected lungs has been reported for some bacterial pathogens, studies in *M*. *tuberculosis*-infected nonhuman primates have shown only limited accumulation in infected lung tissue [148]. Observations in mice appear to suggest a more nuanced picture of MAIT cell contributions to the host control of mycobacterial infection in this model organism. Initial studies indicated contributions of MAIT cells to early host control of mycobacterial infection in the lung upon aerosol or intranasal challenge, as well as in spleen after intravenous delivery of bacteria, albeit with relatively small and transient effects [141,149,150]. In contrast, a recent study using MR1-deficient mice reported no difference in the ability to control *M*. *tuberculosis* infection compared to wild-type mice [151]. Exogenous administration of 5-OP-RU (in conjunction with Toll-like receptor (TLR) agonists) prior to *M*. *tuberculosis* infection resulted in expansion of MAIT cells but did not affect *M*. *tuberculosis* burden in the lung [151,152], despite delayed CD4^+^ T cell priming in mesenteric lymph nodes [151]. On the other hand, therapeutic administration of 5-OP-RU well into the chronic phase of *M*. *tuberculosis* infection conferred some protection in the lung dependent on IL-17A, but not TNF or IFNγ. A possible interpretation of these observations is that the microenvironment and/or activation status of MAIT cells at the time of stimulation skews their cytokine profile towards regulatory or inflammatory functions [151]. Whether exogenous application of MAIT cell antigen would have similar effects in humans will be important to establish, especially considering the relative higher abundance of a MAIT cells in humans when compared to laboratory mice [137]. Such insights will be critical especially if targeted engagement of MAIT cells is to be explored for host-directed interventions in TB [151]. Thus, experimental evidence to date suggests that MAIT cells contribute to host responses against *M*. *tuberculosis* infection, and that it appears to be important to determine whether the timing of their engagement in the context of infection is beneficial or detrimental to immune responses that control mycobacterial infections. Of note, a genetic polymorphism in *MR1* has been associated with TB susceptibility and manifestation in humans [153], and household contact studies have led to the hypothesis that MAIT cells in early stages of *M*. *tuberculosis* exposure are associated with protection from productive infection [147,154]. Findings that abundance or depletion of distinct bacterial species correlates with distinct MAIT cell functionality (e.g., IFNγ, granzyme B expression) in a TB household contact study [147] might be reflective of the impact of phylogenetic diversity, relative demand for riboflavin, and/or carbon source utilization within microbial ecosystems as indicated in in vitro studies on MAIT cell activation [155,156]. Whether these observations translate into in vivo settings with diverse microbial ecosystems at different anatomical sites requires further investment into more detailed analyses on how the microbiome shapes innate immune cell responses at mucosal barriers (Fig 3).

### Adaptive immunity

#### T cells

CD4^+^ T cells are critical in the host control of *M*. *tuberculosis* infection, with contributions of CD8^+^ T and B lymphocytes increasingly appreciated. Inflammatory circuits, e.g., driven by IL-12/IFNγ, TNF, and IL-17, are central to controlling *M*. *tuberculosis*, yet tight regulation of these immune effector mechanisms, e.g., by regulatory T (T_reg_) cells and IL-10, is essential for preventing severe pathology and poor pathogen control [110]. With the growing understanding of how dynamic interactions between microbiota and the host immune system define the development and functions of lymphocytes [157], there is a growing interest in how the microbiota shapes adaptive immune responses that are critical for the host control of *M*. *tuberculosis* infection [44,158].

There is evidence suggesting that microbiota composition licenses T cell functions critical to controlling *M*. *tuberculosis* infection. A recent study in a small cohort of patients with active TB (prior to treatment commencement), LTBI and healthy controls reported a positive correlation between the abundance of Coriobacteriaceae in fecal samples of LTBI individuals with *M*. *tuberculosis* antigen-specific IFNγ responses in peripheral blood [159]. Observations in mice indicate the extent and qualitative impact of antibiotic-induced dysbiosis might differentially impact on immune mechanisms that control *M*. *tuberculosis*. Specifically, impaired host control of *M*. *tuberculosis* in mice exposed to broad-spectrum antibiotics exposure was associated with decreased proportions of IFNγ^+^ and TNF^+^ CD4^+^ T cells alongside an increased percentage FoxP3-positive Treg cells in the spleen [160]. In contrast, mice treated with the narrow-spectrum TB antibiotics isoniazid and pyrazinamide displayed a comparatively slight increase in *M*. *tuberculosis* lung burden at the onset of the chronic phase of infection, which was associated with impaired antimicrobial defense by alveolar macrophages, without impact on the percentages of TB antigen-specific T cells [73]. In both settings, FMT experiments in mice rescued antibiotic-induced impairment of *M*. *tuberculosis* control by the host [73,160]. The impact of broad-spectrum antibiotics on mycobacteria-specific T cell responses has been extended to a vaccine setting in mice with impaired CD4 and CD8 activation, as well as impaired generation of lung-resident and effector memory T cells [161].

There are examples of microbiota species that have been suggested to poise the host towards Th1 responses, including *Klebsiella aeromobilis*, *Klebsiella pneumoniae*, and *Bilophila wadsworthia* [162,163]. Defining if and how specific bacterial groups or species within the microbiota gear *M*. *tuberculosis-*specific T cell responses towards increased effector functions (e.g., IFNγ, TNF) and whether this translates into benefits for the host in controlling *M*. *tuberculosis* might offer opportunities for targeted intervention. This might encompass promotion of a specific microbiota composition but could equally be explored for metabolic capacities of the microbiota that define host immune functions. Microbial products and metabolites, in particular SCFAs, have been established as key mediators of immune-modulatory functions of the microbiota [164]. In this context, the potential contributions of SCFAs such as butyrate have become of particular interest (Fig 3).

Butyrate reduced *M*. *tuberculosis* antigen-specific IFNγ and IL-17A production and elevated IL-10 production of *in vitro* cultured human peripheral blood mononuclear cells (PBMCs) [165,166]. This is consistent with the immune-modulatory functions of butyrate, which are driven by suppression of histone deacetylase (HDAC) activity that enhances *FOXP3* expression and Treg differentiation [167,168]. Additional effects of SCFA on immune functions include reprogramming of Th1 cells towards IL-10 production [169], inhibition of HDAC-dependent epigenetic regulation of inflammatory gene expression (e.g., *IL12b*, *Nos2*) by macrophages and dendritic cells [170,171], as well as limiting neutrophil activation [172]. Thus, the SCFA profile arising from a particular microbiome composition may impair immune effector mechanisms that are central to effective host control of *M*. *tuberculosis*. If present at the time of *M*. *tuberculosis* encounter, this may represent a risk factor for successful infection and progression to active TB. Support for this hypothesis may be drawn from a recent study in a cohort of HIV^+^ healthy individuals undergoing antiretroviral therapy (ART) in a high-TB incidence environment. Individuals undergoing ART are characterized by SCFA-producing microbiota in their lower airways, and in this cohort, SCFA serum concentrations positively correlated with elevated risk of subsequently developing TB, as well as induction of FoxP3^+^ Tregs in PPD-stimulated cultures of BAL lymphocytes [165]. Elevated serum SCFA concentrations were associated with increased presence of *Prevotella* in the lower airways [165]. These correlations encourage investigation of how SCFA production locally in the lung, or systemically, might hamper mucosal immune defense mechanisms against *M*. *tuberculosis* infection. This might seem counterintuitive when considering the decrease in the relative abundance of Ruminococcaceae and/or Lachnospiraceae described in some studies (Table 1 and Fig 1A). However, if altered microbiota composition in the context of active TB disease was accompanied by diminished SCFA concentrations at peripheral sites, one might speculate that microbiota changes upon *M*. *tuberculosis* infection could be reflective of a directly or indirectly driven host adaptation to enable effective Th1 immune responses that control *M*. *tuberculosis*. Carefully designed longitudinal studies, integrating taxonomic, metagenomic, metabolomic, and immunological analyses in a prospective setting will be necessary to establish whether a microbiome composition functionally geared towards a specific metabolic output governs establishment and host control of *M*. *tuberculosis* infection.

#### B cells and antibodies

Mucosal and systemic antibody responses are directly shaped by the microbiome. Exploration of these microbiota-immune interactions has largely focused on the gut microbiota, a critical regulator of gut immunoglobulin A (IgA) production [173,174]. Microbiota-derived SCFAs gear B cell metabolism and gene expression towards antibody production [175]. TLR-mediated sensing of the microbiota by epithelial and dendritic cells drives expression of a proliferation-inducing ligand (APRIL) and B cell-activating factor (BAFF), which promote B cell survival and IgA production by plasma cells [176–180]. There is emerging evidence that microbial cues at oral and respiratory epithelial sites similarly shape B cell functions and antibody responses [180–182]. Despite these well-established links between microbiota and antibody responses, it remains largely unknown how these contribute to host responses during *M*. *tuberculosis* infection and TB disease.

The B cell compartment in peripheral blood undergoes dynamic changes during *M*. *tuberculosis* infection, and relative abundance of memory B cells, plasma blasts, and plasma cells has been correlated with TB disease state (reviewed in [183]). *M*. *tuberculosis* infection induces robust antibody responses, yet the contributions of B cells to the immune control of the infection are incompletely understood and have remained controversial. Different mouse models of B cell deficiency indicated protective contributions of B cells during *M*. *tuberculosis* infection, through regulation of tissue pathology and local inflammatory cytokine responses [184–186]. B cell depletion (anti-CD20, rituximab) in *M*. *tuberculosis*-infected nonhuman primates did not affect overall lung pathology, bacterial burden, and clinical outcome in an early disease setting. Nevertheless, at the level of individual granulomas, B cell contributions to bacterial control, production of IL-6 and IL-10, as well as diminishing the frequency of T cells expressing IL-2, IL-10, or IL-17 have been reported [187].

*M*. *tuberculosis* infection in the immune-competent host elicits robust antibody responses against diverse mycobacterial protein and oligosaccharide antigens [188]. Recent insights into potential roles of antibody-mediated modulation of *M*. *tuberculosis* control by host cells [189,190] have reinvigorated the interest in B cell functions in TB. Antibody-mediated opsonization (serum or purified IgG) has been implicated in *M*. *tuberculosis* restriction by infected human and mouse macrophages associated with enhanced phagocytosis and delivery to phagolysosomal compartments [189–194]. More detailed insights into patient-specific patterns and functional contributions of IgG subtypes in this context will be of great value, especially in light of earlier observations implicating distinct outcomes of activating versus inhibitory Fcγ receptors for the host control of *M*. *tuberculosis* infection [195]. Antibiotics-mediated depletion of resident microbiota has been associated with decreased pulmonary IgA production, which has been associated with increased susceptibility to pulmonary bacterial infections in humans and mice [180]. This observation likely bears relevance for *M*. *tuberculosis* infection in light of reports that passive transfer of purified, mycobacteria-specific IgA diminished bacterial burden in infected lungs [196–198]. The molecular and cellular mechanisms underlying this protection are incompletely understood but may include IgA-mediated inhibition of infection of macrophages and lung epithelial cells with contributions of the human FcαRI IgA receptor [190,198]. Humoral immune responses in individuals infected with *M*. *tuberculosis* are highly heterogenous and influenced by complex interactions of a number of factors, including age, state of infection (active TB disease or LTBI), and immune competency (e.g., HIV, diabetes). With the fundamental contributions of the microbiota to shaping local airway mucosal as well as systemic antibody responses [173,199], it is imperative to define how microbiota-defined local and systemic antibody responses affect host susceptibility and manifestation (active disease versus LTBI) during *M*. *tuberculosis* infection. The design of future studies needs to include considerations on the impact of systemic and mucosal antigen exposure on antibody repertoire [199]. Isotype- and/or target cell-specific functional differences of *M*. *tuberculosis*-specific antibodies may be further defined by distinct glycosylation profiles characteristic to disease state, i.e., active versus latent TB [189]. It will be important to determine whether treatment with TB antibiotics causes secondary IgA deficiency [180] and whether this poses risks for (re)infection with *M*. *tuberculosis*. A comprehensive view of B cell functionality, beyond antibody responses, in this context will further enhance understanding of cellular drivers of local inflammatory responses [185,187], macrophage polarization [200], neutrophilia [185,201], and immune regulation [202,203].

## Are there opportunities for microbiota-focused preventative and adjunct-therapeutic strategies?

With the notion that the larger collective of “commensal microorganisms” may, directly and indirectly, shape host susceptibility to *M*. *tuberculosis* (re)infection, protective immune responses, and disease manifestation, the questions arising now center on how this knowledge might translate into therapeutic or preventative measures. Areas of focus include opportunities at the gene product (e.g., metabolites and bioactives), organismal (e.g., probiotics, genetically modified organisms (GMO), FMTs), and dietary level of interventions to correct microbial dysbiosis or specifically deliver functional capabilities that reshape host immune responses and resilience to *M*. *tuberculosis* infection and/or recurrence.

Strategies that promote the introduction and/or restoration of a “beneficial” microbiota, such as dietary interventions or defined probiotic formulations may prove to be an effective strategy to complement TB treatment, in particular in correcting the long-lasting dysbiosis that occurs as consequence of prolonged TB antibiotics regimens. Moreover, gut microbiota composition prior to infection has been found to correlate with disease manifestation in nonhuman primates experimentally infected with *M*. *tuberculosis*, which raises the possibility of defining a gut microbiota that reduces host susceptibility to *M*. *tuberculosis* infection and TB disease manifestation [204]. Gut microbiota diversity, abundance, and host immune response are strongly impacted by diet and nutrition and much still needs to be learned about these interrelationships in the context of disease susceptibility and prevalence associated with under-, mal-, and overnutrition [120]. Protein–calorie undernutrition, type 2 diabetes associated with overnutrition, and micronutrient deficiencies (e.g., vitamin D) are risk factors for developing active TB [205–208].

Probiotics such as *Bifidobacterium* spp. as an adjunct therapy with conventional TB antibiotics are reported to restore and maintain what is considered a “healthy microbiome” [209–211]. A longitudinal study in TB patients reported that a multi-strain probiotic formulation (*Lactobacillus acidophilus*, *Lactobacillus casei*, *Lactobacillus rhamnosus*, *Lactobacillus bulgaricus*, *Bifidobacterium breve*, *Bifidobacterium longum*, and *Streptococcus thermophilus*) combined with supplementation of vitamins B_1_, B_6_, and B_12_ increased serum concentrations of IFNγ and IL-12, compared to the control group receiving only anti-TB antibiotics and vitamin B_6_ [212]. Whether rational design of safe-for-human-use probiotics can include the design of strains that withstand TB antibiotic therapy as proposed recently [213] remains to be carefully evaluated.

Immune cross-reactivity between mycobacterial species as well as direct impact on the microbiota are associated with beneficial effects of orally administered heat-killed *Mycobacterium manresensis*. Indeed, formulations using this environmental bacterium that is commonly found in drinking water are being explored for potential benefits in the treatment of TB. In a susceptible mouse model of *M*. *tuberculosis* infection, orally administered heat-killed *M*. *manresensis* reduced lung pathology, bacterial burden, and inflammatory responses, and in combination with TB antibiotics, expanded the life span of infected mice when compared to mice treated only with antibiotics [214]. Following on from early clinical safety profiling [215,216], a placebo-controlled randomized interventional trial in HIV–negative and HIV–positive individuals undergoing treatment for TB is currently analyzing the impact of a *M*. *manresensis*-based food supplement on gut microbiota composition, antigen-specific CD4+ T cell responses, as well as time to sputum conversion and reduction in bacterial burden (NCT03851159).

Perhaps the most dramatic approach to “probiotic therapy” is the integration of FMT into clinical practice. Although practiced by some cultural groups for centuries [217], FMT has recently become a mainstream intervention for the treatment of recurrent *Clostridioides difficile* infection, offering high therapeutic efficacy and with limited prevalence of adverse events, at least in the short term [218]. These findings have catalyzed global interest in both research and clinical settings for the evaluation of FMT as induction therapy for a variety of medical conditions where gut “dysbiosis” is implicated [219–221]. In the context of TB, the findings that FMT reversed the increased susceptibility of antibiotic-treated mice to *M*. *tuberculosis* infection [73,160] warrants further investigation into microbiota compositions that confer benefits to the host. In summary, probiotics as an adjunct and/or therapeutic option for the restoration of gut homeostasis has long been investigated and continues to hold promise, and this extends to their potential as adjunct therapeutics alongside TB antibiotics [222–224].

With current limitations of probiotics and FMT, dietary interventions, defined microbial metabolites, and actively secreted bioactives might offer a pragmatic alternative. For example, indolepropionic acid (IPA), which is produced by bacteria taxonomically affiliated with the Clostridiales, including *Peptostreptococcus anaerobius*, has been shown to inhibit growth of *M*. *tuberculosis*, both in vitro and in vivo. This has been attributed to antagonistic effects of IPA on *M*. *tuberculosis* tryptophan biosynthesis, leading to suggestions that IPA per se and/or targeting the *M*. *tuberculosis* tryptophan pathway may be avenues for the discovery of novel antimycobacterials [225–228]. Additional positive effects of IPA on epithelial barrier function as well as activation of innate and adaptive immune responses [229–232] might be worth exploring for dually acting compounds. A second example are bacteria-derived AMPs, which directly affect microbial ecology, including specific inhibition of bacterial pathogens [233,234]. The in vitro antimycobacterial activity of bacteriocins isolated from *Lactobacillus salivarius*, *Streptococcus cricetus*, and *Enterococcus faecalis* exceeds that of the TB antibiotic rifampicin [235], with nisin and lacticin being effective towards *M*. *tuberculosis*, *Mycobacterium kansasii*, *Mycobacterium smegmatis*, and *Mycobacterium avium* subspecies *paratuberculosis* [236,237]. Synergism with TB antimicrobials, such as those reported for bacteriocin AS-48 from *E*. *faecalis* and ethambutol [238] may offer avenues for exploration, e.g., whether combinations allow for shortening of current antibiotics regimens or reducing antibiotic dosing to limit toxic side effects.

Notwithstanding the notion that SCFAs poise host immune mechanisms towards a permissive environment for *M*. *tuberculosis* infection, whether modulation of SCFA production might be a target for intervention in TB requires careful consideration. With SCFA the primary microbial metabolites released within the gastrointestinal tract, host evolution has favored the development of sensor-regulatory pathways linked with immune and/or metabolic pathways that can monitor and respond to alterations in these primary microbial metabolites. In chronic diseases with characteristic gut dysbiosis (e.g., inflammatory bowel disease), the presumptive reduction in butyrate-producing bacteria is widely considered to compromise barrier integrity, mucin production, and FoxP3^+^ Treg cell production [239,240]. While the link between SCFAs and host immune responses is relatively well characterized, the minimal effective concentrations of SCFA needed for the maintenance of barrier integrity and regulatory immune responses are less well understood. In that context, the therapeutic efficacy of specifically modulating colonic butyrate and/or other SCFA concentrations via oral or colonic routes of administration are, at best, mixed [241]. Such findings suggest that reaching threshold concentrations of colonic SCFA are necessary but not sufficient to bias mucosal integrity and immune responses. Indeed, additional metabolic capabilities being defined in “beneficial” bacteria such as *Faecalibacterium prausnitzii* [242,243] highlight the complexity of microbial metabolites and secreted products that define the sustainability of gut homeostasis and poise (mucosal) immune responses.

## Conclusions

Confidence in whether the microbiome composition is associated with host susceptibility *M*. *tuberculosis* infection or can indeed skew effector mechanisms towards improved or diminished pathogen control requires carefully designed prospective and longitudinal studies in large cohorts. The integration of microbiome, metagenome, and metabolome analyses, ideally in the lung as well as the gut and potentially other distant sites, alongside immunological characterization will be essential. Additionally, important confounding factors such as nutritional status, coinfection(s), and other comorbidities [165,244] will need to be integrated into study and cohort design. Careful considerations will need to be given to sampling techniques, as well as appropriate control samples and cohorts [8].

Candidate microbiota/microbe/metabolite approaches and functional studies in animal models of TB will be invaluable to further elucidate causality between microbiota composition, metabolic capacity, and the immune control of *M*. *tuberculosis* infection. It will be particularly important to determine the interplay between microbiota and immune components at distinct stages of infection and disease. Our discussions above highlight the importance of acknowledging potential composite effects of innate and adaptive immune cell functions, and the multidimensional interplay between microbiota and host defense mechanisms. For example, butyrate enhanced antimicrobial defense in macrophages (e.g., AMP expression and autophagy), thereby increasing control of extracellular and intracellular bacterial pathogens, including mycobacteria [245]. Yet, SCFAs are emerging to create a permissive immune milieu for *M*. *tuberculosis* infection in the host at least in part through their immune-modulatory effects on adaptive immune responses. Moreover, detailed studies are required to fill current knowledge gaps on the host interactions with viruses, fungi, and protozoa in the human microbiome, which likely has profound implications for shaping host responses to infections [246,247].

Restoration of TB antibiotic-induced dysbiosis is an attractive and seemingly achievable target. Nevertheless, the transition of probiotics from being dietary supplements to an evidence-based predictive intervention in clinical settings remains elusive [248,249]. Similarly, the potential that FMT might serve to augment the treatment and immune control of *M*. *tuberculosis* infection, as indicated in mouse studies [73,160], is attractive. With the accelerating increase in reports associating microbiota composition with human pathologies, some level of caution is warranted, e.g., in relation to invariably positive outcomes from studies using human microbiota-associated or humanized gnotobiotic animal models [61]. Additional critical considerations need to be given to the ethical, cultural, and safety implications of selecting and using stool samples for FMT, which continue to be reviewed and assessed for other conditions where gut dysbiosis is diagnostic [250]. Similarly, interest in using diet as a first-line intervention for the correction of microbiota-immune interactions and promoting gut homeostasis in digestive health and disease have gained considerable momentum in recent years [251,252]. Translation of these findings to the context of TB may offer insights over and above gains made by promoting a more protein–calorie-rich diet in societies afflicted by mal- and/or undernutrition. But not unlike the constraints associated with the advancement of probiotics, FMT, and next-generation versions of both, the translation of such observations into evidence-based interventions is contingent on further refinement of the approaches used to produce such evidence [253].

In summary, notwithstanding the increasing body of literature focused on establishing links between the microbiome and the immune control of TB, as with most microbiome-focused research, the challenge at hand will be to establish causality, which would deliver solid foundations for the pursuit of targeted interventions in TB.
